# Tuberculosis of the Thyroid in a Child: A Rare cause of Thyromegaly 

**Published:** 2016-01-01

**Authors:** Sugandha Sharma, Manish Girhotra, Naushad Zafar, Ruchika Gupta

**Affiliations:** 1 Department of Pathology, Chacha Nehru Bal Chikitsalaya, Geeta Colony, Delhi – 110031, India; 2 Department of ENT, Chacha Nehru Bal Chikitsalaya, Geeta Colony, Delhi – 110031, India

**Keywords:** Tuberculosis, Thyroid, Pediatric, Aspiration cytology

## Abstract

Tuberculosis of thyroid gland is extremely rare in children. We describe the case of a 6-year girl child, presenting with a multinodular thyroid swelling. Fine needle aspiration cytology showed extensive necrosis with few epithelioid cell granulomas and occasional acid-fast bacilli, suggesting a diagnosis of tuberculosis. The child was put on anti-tuberculous drugs with significant improvement. Tuberculosis of the thyroid gland, although rare, should be considered in differential diagnosis of thyroid swelling, especially in endemic areas.

## INTRODUCTION

Tubercular thyroiditis is a rare disease in pediatric population.[1,2] Thyroid tuberculosis (TTB) is usually not suspected clinically due to absence of distinctive features. Majority of the cases have been diagnosed on either cytological or histopathological examination.[3] We report the clinico-pathologic features of multinodular thyroid swelling in a girl.

## CASE REPORT

A 6-year-old female child presented with midline neck swelling for the past two months. She also had low-grade intermittent fever. There was no history of associated difficulty in swallowing or breathing. No features suggestive of hypo- or hyper-thyroidism were present. On local examination, there was multinodular thyroid swelling. The nodules were variable in size, largest being 2 cm x 2 cm. The nodules were soft to firm in consistency and slightly tender. The swelling moved up with deglutition. There was no fixity to the overlying skin or underlying structures. Laboratory investigations, including thyroid function tests, were within reference range.

On ultrasonography (USG), thyroid gland was diffusely enlarged with altered echotexture. Multiple ill-defined and few well defined nodules were seen in both lobes. Largest nodule in right lobe measured 11mm x 8 mm and that in the left lobe measured 15 mm x 3mm. A radiological impression of multinodular goiter was made. On fine needle aspiration yellowish material was obtained. Giemsa-stained smears showed large areas of caseous necrosis with few epithelioid cell granulomas and foamy histiocytes (Fig. 1a and b). Scattered clusters of benign follicular cells were also seen (Fig. 1c). Ziehl-Neelsen stain revealed occasional acid-fast bacilli (Fig. 1d). A final cytological diagnosis of tuberculous thyroiditis was made.

**Figure:**
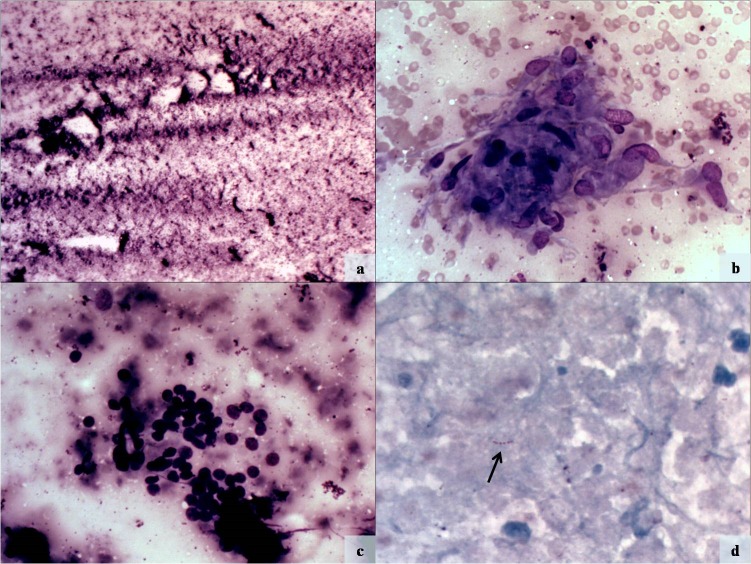
Figure 1:Photomicrographs showing extensive caseous necrosis (a, Giemsa x100) and few epithelioid cell granulomas (b, Giemsa x200). A group of benign follicular epithelial cells is seen in c (Giemsa x200). Ziehl-Neelsen stain demonstrates acid-fast bacillus (arrow) in necrotic background (d, x1000).

The child was investigated further with Mantoux test, chest roentgenogram and abdominal USG. All were reported as within normal range. Anti-tuberculous therapy with four drugs for three months followed by consolidation phase of three drugs for six months was advised. A follow-up examination at six months showed significant reduction in the size of swelling.

## DISCUSSION

The exact incidence of TTB varies from 0.2% in chronic thyroiditis to 7% in miliary tuberculosis.[4] This rarity of occurrence has been attributed to bactericidal property of colloid, high vascularity, iodine excess and possible role of thyroid hormones.[4] Most of the cases of TTB have been reported in middle-aged females. Our patient was six year of age. Thyroid function tests usually remain within reference ranges in patients with TTB, as was seen in our patient. Occasionally, thyrotoxicosis or hypothyroidism occurs in association with TTB. [5]

Fine needle aspiration cytology is effective in the diagnosis of TTB.[6] The characteristic cytological finding of epithelioid cell granulomas with caseous necrosis and acid-fast bacilli makes the diagnosis certain.[3] In the present case also, caseous necrosis with granulomas and occasional aid-fast bacilli were seen.

Complete resolution of TTB is possible with appropriate and adequate duration of anti-tuberculous drug treatment. Surgical drainage or resection may be needed in patients with large abscess.[6] Thyroid tuberculosis is extremely rare in pediatric age group. It may be considered in the differential diagnosis of thyroid masses especially in endemic countries.

## Footnotes

**Source of Support:** Nil

**Conflict of Interest:** None declared

